# The effects of increasing dosages of narasin on ruminal fermentation patterns, bacterial community composition, and nutrient digestibility in beef cattle receiving feedlot diets

**DOI:** 10.1371/journal.pone.0346130

**Published:** 2026-04-02

**Authors:** Ana Laura Januário Lelis, Leandro Aparecido Ferreira da Silva, Daniel Moretto Casali, Tiago Leiva, Murilo Chuba Rodrigues, José Paulo Roman Barroso, Pedro Veloso Facury Lasmar, Camila Lisboa Tomaz, Anabelle Jorge Barbosa, Camila Cesario Fernandes Sartini, Johnny Maciel de Souza, Danilo Domingues Millen

**Affiliations:** 1 School of Veterinary Medicine and Animal Science, São Paulo State University (UNESP), Botucatu, São Paulo, Brazil; 2 Elanco Animal Health, São Paulo, São Paulo, Brazil; 3 School of Agricultural and Veterinary Sciences, São Paulo State University (UNESP), Jaboticabal, São Paulo, Brazil; 4 School of Agricultural and Technological Sciences, São Paulo State University (UNESP), Dracena, São Paulo, Brazil; University of Illinois, UNITED STATES OF AMERICA

## Abstract

This study evaluated the effects of increasing narasin doses on ruminal fermentation, nutrient digestibility, ruminal pH stability, papillae histology, and microbial composition in Angus cattle fed feedlot diets. Three rumen-cannulated Angus steers (average body weight: 680 kg) were assigned to a 3 × 3 Latin square design and received diets containing 13, 20, or 27-ppm of narasin. Each experimental period consisted of 14 days of adaptation followed by seven days of sampling. Ruminal degradability was assessed on days 15–17, apparent digestibility on days 15–19, continuous ruminal pH on days 19–20, and samples for short-chain fatty acids (SCFA), microbiota, and ruminal histology were collected on days 20 and 21. Ruminal degradability was not affected by narasin dose. Digestibility of acid detergent fiber (ADF) was significantly influenced, with the greatest values observed at 27-ppm (*P* = 0.01). Increasing narasin doses improved ruminal pH stability, as indicated by a linear increase in minimum pH (*P* = 0.01) and a reduction in the duration of pH below 5.6 (*P* = 0.10). At 13 ppm, SCFA production, particularly acetate and propionate, increased (*P* < 0.05), indicating enhanced fermentation efficiency. In contrast, supplementation with 27-ppm reduced ammonia (*P* < 0.01), acetate (*P* = 0.02), and butyrate (*P* < 0.01) concentrations and increased the acetate-to-propionate ratio (*P* < 0.01). Lactate concentration decreased linearly with increasing narasin doses (*P* = 0.03). Narasin supplementation altered ruminal microbial composition, increasing the relative abundance of Lachnospiraceae and *Isotricha* while reducing lactic acid–producing bacteria. In terms of ruminal morphology, supplementation with 20-ppm of narasin increased the keratin layer thickness of ruminal papillae (*P* = 0.02), suggesting enhanced epithelial development. Overall, narasin supplementation modulated ruminal function and microbial ecology, with doses between 13 and 20-ppm providing the most favorable balance between fermentative efficiency and ruminal health in feedlot cattle.

## Introduction

Most livestock production in Brazil occurs in pasture-based systems; however, feedlot systems have become increasingly popular for finishing cattle more quickly and improving feed efficiency. In these systems, the average inclusion level of concentrate ingredients in the diets is 83.5% [1], with corn being the primary ingredient used. Improving production efficiency in ruminants requires optimizing ruminal fermentation, enhancing the quality of dietary ingredients, or manipulating microbial populations in the rumen [2].

However, when feeding diets containing high levels of quickly fermentable carbohydrates, significant ruminal changes may occur, including increased free glucose availability, stimulation of bacterial growth, elevated production of short-chain fatty acids (**SCFA**) and lactic acid, reduced ruminal pH, and decreased rumen motility [3]. As preventative micro-ingredients, feed additives are commonly included in feedlot diets to reduce digestive disorders, such as acidosis, and to optimize ruminal fermentation. Among these additives, ionophores are widely used [1], including monensin, one of the most studied ionophores that has demonstrated the ability to enhance SCFA production, alter their proportions, reduce dry matter intake (**DMI**), and, in some cases, improve animal performance [4]. However, narasin, an ionophore originally studied in cattle consuming high-forage diets, has been shown to increase average daily gain (**ADG**) without affecting DMI, and at a lower dose than monensin [5–8]. Both monensin and narasin are polyether compounds, and the arrangement and specific types of functional groups, particularly hydroxyl and carboxyl groups, differ between the two molecules, influencing their shape and interaction with ions.

Narasin seems to be a promising ionophore for feedlots that employ diets similar to those fed in Brazil (1.27 vs. 1.50 Mcal of net energy for gain/kg of dry matter in USA [**DM**; Silvestre and Millen [1]; Samuelson et al. [9], respectively]. In feedlots in the U.S. and Brazil, monensin is commonly used to enhance feed efficiency and control and prevent coccidiosis [9–11]; however, due to the lower energy content of finishing diets in Brazil, the use of an ionophore that does not affect DMI, such as narasin, may be desirable. A dose of 13 ppm of narasin is recommended for cattle grazing on or fed high-forage diets [5].

In addition to its effects on ruminal fermentation, narasin has been shown to present a wide safety margin in ruminants when administered within recommended inclusion levels. Toxicological evaluations indicate that adverse effects are primarily associated with overdoses or use in non-target species, whereas cattle exhibit high tolerance to narasin within the approved inclusion range [12,13]. Thus, when properly dosed, narasin is considered safe for use in beef cattle diets, supporting its evaluation as a feed additive in feedlot systems [12].

Therefore, the dosage of narasin tested in this experiment was based on the most effective responses observed in studies involving high-forage diets for beef cattle. However, the limited research on narasin in high-concentrate diets raises questions about its efficacy and optimal dosing for feedlot cattle. Thus, we hypothesize that increasing levels of narasin will positively influence ruminal metabolism in Angus cattle, resulting in beneficial changes to ruminal fermentation patterns and bacterial community composition. The objective of this study was to evaluate the effects of increasing narasin doses on ruminal fermentation, microbial community composition, and nutrient digestibility in rumen-cannulated Angus cattle fed feedlot diets.

## Materials and methods

### Animals and experimental facility

Animal care and handling used in this experiment adhered to the guidelines of the Animal Use Ethics Committee (CEUA) and were approved by the Ethics Committee on Animal Use of São Paulo State University (UNESP), Dracena campus, Brazil (Protocol CEUA 003/2022).

The study was conducted at the São Paulo State University (UNESP) feedlot, Dracena campus, Brazil. Three rumen-cannulated Angus steers of an approximately 36 months of age and with an initial body weight of ± 680 kg, were utilized in the study.

### Experimental design

The experimental design, a duplicated 3 × 3 Latin square with three rumen-cannulated Angus steers rotating across treatments, was chosen to maximize the use of available animals while controlling for period and carryover effects. It means that each animal received each treatment twice throughout the study and was used in both squares. Based on variability estimates from previous studies with similar parameters, such as ruminal pH, SCFA concentrations, and nutrient digestibility, the study design provides an approximate statistical power of 0.80 (80 percent) to detect biologically relevant differences among treatments at a significance level of 0.05. This power estimation indicates a high likelihood of detecting meaningful treatment effects, ensuring the robustness of the observed responses to narasin supplementation. The experimental treatments consisted of three levels of narasin supplementation: 13, 20, and 27 ppm per kilogram of dietary dry matter. A negative control group, 0-ppm narasin, was not included in this study because of the limitation on cannulated animals, as well as because we included the negative control treatment in the companion paper on feedlot performance [14].

The 13-ppm treatment is commonly used in supplements for grazing systems [6,15,16]. Since feedlot diets have a higher energy content than forage diets, the tested doses were equal to or greater than the typical dose for grazing animals.

### Management, feeding, and animal handling

Before the experiment, a single 5-day adaptation period was implemented to acclimate animals to the facilities, handling procedures, and experimental conditions. This adaptation period occurred only once, prior to the first experimental period.

Each experimental period lasted 21 days, comprising 14 days of dietary adaptation to the finishing diet followed by 7 days on the finishing diet, during which sampling was performed, resulting in a total experimental duration of 156 days. The dietary adaptation within each experimental period followed a standardized step-up protocol and was identical across all periods and treatments. This protocol consisted of three sequential diets, herein referred to as adaptation 1, adaptation 2, and adaptation 3.

Adaptation 1 consisted of a diet containing 75% concentrate and was fed for 5 days. Adaptation 2 contained 79% concentrate and was fed for 4 days. Adaptation 3 contained 83% concentrate and was fed for 5 days. The finishing diet consisted of 87% concentrate ingredients (Table 1). Tifton hay was used as the sole forage source and was gradually reduced across the adaptation diets.

**Table 1:** Feed ingredients and chemical composition of the experimental diets provided to rumen-cannulated Angus cattle during the adaptation and finishing phases.

Diets	Adaptation 1	Adaptation 2	Adaptation 3	Finishing
**Concentrate (%)**	75.0	79.0	83.0	87.0
**Ingredients (% DM)**				
Tifton hay	15.0	11.0	7.0	3.0
Peanut hulls	10.0	10.0	10.0	10.0
Finely-ground corn	51.0	56.0	61.0	66.0
Soybean meal	10.0	8.0	6.0	4.0
Cottonseed cake	11.0	12.0	13.0	14.0
Urea	0.5	0.5	0.5	0.5
Mineral supplement	2.5	2.5	2.5	2.5
**Nutritional Content (% DM)**				
TDN^1^	78.0	80.0	81.0	82.0
Crude protein	15.8	15.1	14.4	13.8
Ether extract	4.0	4.1	4.2	4.3
Neutral detergent fiber	33.9	31.7	29.6	27.5
peNDF^2^	23.0	21.0	18.0	16.0
NE_g_, Mcal/kg	1.12	1.15	1.19	1.23
Ca	0.52	0.50	0.48	0.47
P	0.41	0.41	0.41	0.42

^1^ Total digestible nutrients, ^2^Physically effective neutral detergent fiber.

A 5-day washout period was included between consecutive experimental periods to minimize carryover effects. During washout, animals were released to pasture and did not receive experimental diets; therefore, washout periods were not considered part of the adaptation protocol.

Feeding occurred once daily in the morning at 08:00. The amount of diet provided was adjusted daily based on the refusals from the previous day and managed to maintain refusals at approximately 1% of feed offered, aiming to minimize feed sorting and ensure consistent intake of the narasin. Diets and refusals were collected and stored for chemical analysis. Narasin was supplied during both the dietary adaptation phases and the finishing phase, maintaining the exact dosages established for each treatment.

The experimental diets were formulated according to the LRNS (Large Ruminant Nutrition System, level 2). Feed ingredients were mixed in a truck-mounted mixer. Narasin was administered using soybean meal as a carrier, applied on top of the diet, and mixed immediately after being placed in the bunk feeder.

Experimental diets and ingredients were sampled weekly, as outlined by Pereira et al. [17], to determine DM [18] (method 930.15), ether extract (method 920.39), crude protein (method 990.02), neutral detergent fiber (NDF) [19], and ash (method 942.05).

### Total dry matter and nutrient digestibility

The total nutrient digestibility (DM, CP, NDF, ADF, starch, and ash) was determined using titanium dioxide (TiO_2_) as an external marker [20,21]. Titanium dioxide was administered via the ruminal cannula at a rate of 1 g/kg of diet DM from days 10–19 of each experimental period, with half of the daily dose provided at 08:00 and the remainder at 16:00.

From days 15–19, samples of feces, diets, and feed refusals were collected daily. At the end of the collection period, samples were dried and ground through a 2-mm sieve, and pooled to form a composite sample representing the five days of collection.

Apparent digestibility coefficients were calculated based on the concentrations of TiO_2_ and nutrients in the diet, refusals, and feces, according to the following equation:


$$\text{Digestibility }(\%)\;=\text{ 100 }\times \;[\text{1}\;-\;({\text{TiO}}_{\text{2}}\_\text{feed }\times \text{ Nutrient}\_\text{feces})/\;({\text{TiO}}_{\text{2}}\_\text{feces }\times \text{ Nutrient}\_\text{feed})],$$

where TiO_2__feed and TiO_2__feces represent titanium dioxide concentrations (g/kg DM) in feed and feces, respectively, and Nutrient_feed and Nutrient_feces represent nutrient concentrations (g/kg DM) in feed and feces, respectively.

### Ruminal degradability in situ

Ruminal in situ degradability was evaluated on days 15, 16, and 17 of each experimental period according to the methodology described by Mehrez and Ørskov [22]. Nylon bags (50-µm pore size; 100 × 190 mm) were filled with approximately 25 g of the experimental diets, previously dried at 65°C for 72 h.

Bags were incubated in the rumen for 0, 6, 12, 24, 48, and 72 h. For each incubation time, three replicate bags were used per animal. After removal from the rumen, bags were washed under running water until the rinse water was clear, and subsequently dried at 65°C for 72 h. Residues were analyzed for starch [23], crude protein (CP), neutral detergent fiber (NDF), acid detergent fiber (ADF) [19], and ash.

Degradability parameters were estimated using the model proposed by Ørskov and McDonald [24], and potential and effective degradability were calculated according to Ørskov et al. [25]. Apparent nutrient degradability was calculated using the following equation:


Degradability (%) = 100 × [(weight after incubation − empty bag weight)/ (weight before incubation −empty bag weight)].

### Continuous ruminal pH measurement

Continuous ruminal pH, temperature, and redox potential were recorded on days 19 and 20 of each experimental period using the Lethbridge Research Centre pH system (LRCpH) equipped with a T7-1 data logger (Dascor, Escondido, CA, USA). Measurements were obtained at 5-min intervals. Two 900-g weights were attached to the probe to ensure consistent contact with the ruminal epithelium [26].

Data were used to calculate minimum, mean, and maximum ruminal pH, as well as the duration (min) that pH remained below 5.2, 5.6, and 6.2, and the corresponding areas under the pH curves, according to Bevans et al. [27]. Prior to each measurement period, the pH sensor was calibrated using standard buffer solutions at pH 4.0 and 7.0.

### Evaluation of ruminal fermentation products

Ruminal fluid samples were collected on day 20 of each experimental period at 0, 4, 8, and 12 h after feeding, with the 0-h sample obtained immediately before feed delivery. At each sampling time, ruminal contents were collected from nine locations within the rumen (three sites from each ruminal stratum) to obtain representative samples. The contents were squeezed through cheesecloth, and approximately 50 mL of ruminal fluid was recovered and transported to the laboratory for analysis.

During sample preparation, 25 mL of ruminal fluid was centrifuged at 3,500 rpm for 15 min, and the resulting supernatant was used for determination of short-chain fatty acids (SCFA), lactic acid, and ammonia nitrogen (NH_3_-N). For SCFA analysis, 2 mL of supernatant was transferred to test tubes, acidified with 4 mL of formic acid, sealed, and stored at −20°C until analysis. Concentrations of acetate, propionate, and butyrate were determined by gas chromatography (Finnigan 9001, Thermo Scientific, West Palm Beach, FL, USA) using a glass column (1.22 m × 0.63 cm) packed with 80/120 Carbopack B-DA/4% (Supelco, Sigma-Aldrich, St. Louis, MO, USA), following the method described by Erwin et al. [28]. Total SCFA concentration was calculated as the sum of acetate, propionate, and butyrate.

Lactic acid concentration was determined using the colorimetric method described by Pryce [29]. For ammonia nitrogen determination, 2 mL of supernatant was mixed with 1 mL of 1 N sulfuric acid and stored at −20°C until colorimetric analysis, according to Kulaseq [30].

### Ruminal bacterial community sequencing

On day 20 of each experimental period, 4 hours after feeding, 50 mL of ruminal content (solid and liquid) was collected from nine points within the rumen, three points from each rumen stratum, composing a representative sample of the ruminal content. A total of 50 mL was placed in sterile tubes and frozen at −80°C. Total DNA was extracted from the ruminal samples using a bead-beating mechanical disruption method followed by phenol extraction, as described by Weimer et al. [31]. The extracted DNA was resuspended in 10 mM Tris-HCl with 1 mM EDTA (pH 8.0), quantified fluorometrically using a Qubit Fluorometer (Invitrogen, San Diego, CA, USA), and stored at 4°C before library preparation. Universal primers targeting the V4 region of the bacterial 16S rRNA gene were used (F: GTGCCAGCMGCCGCGGTAA; R: GGACTACHVGGGTWTCTAAT), as described by Kozich et al. [32]. Primers included unique barcodes for multiplexing and adapters suitable for Illumina sequencing (F: AATGATACGGCGACCACCGAGATCTACAC;CAAGCAGAAGACGGCATACGAGAT). PCR reactions contained 25–50 ng of DNA, 10 μM of each primer, 12.5 μL of 2X KAPA HotStart ReadyMix (KAPA Biosystems, Wilmington, MA, USA), and nuclease-free water to a final volume of 25 μL. Each reaction was performed in duplicate. Cycling conditions were initial denaturation at 95°C for 3 min; 25 cycles of 95°C for 30 s, 55°C for 30 s, 72°C for 30 s; and a final extension at 72°C for 5 min. No-template negative controls were included to confirm the absence of contamination. The PCR products (25 μL) were run on 1% low-melt agarose gels with 5 μL of 6X Orange loading dye. Bands at ~380 bp were excised and purified using a ZR-96 Zymoclean Gel DNA Recovery Kit (Zymo Research, Irvine, CA, USA). DNA was equimolarly pooled and sequenced using MiSeq v2 2 × 250 kit (Illumina, San Diego, CA, USA) with 10% PhiX control, using custom sequencing primers [32]. Sequences were demultiplexed using sample-specific indices on an Illumina MiSeq and deposited in the Short Read Archive of the National Center for Biotechnology Information under BioProject Accession PRJNA1337894.

Sequences were demultiplexed and processed using mothur v1.41.1 [33]. Paired-end reads were merged into contigs; low-quality sequences (ambiguous bases, homopolymers >8 bp, sequences <200 bp) were removed. Sequences were aligned to the SILVA v132 reference database, pre-clustered (diffs = 2), and chimeras removed using UCHIME. Taxonomic classification was performed using the GreenGenes database (August 2013 release) with a bootstrap cutoff of 80. Sequences assigned to Cyanobacteria, mitochondria, Eukarya, or Archaea were removed. Singletons were removed to streamline analysis. Alpha diversity was calculated using the Chao richness estimator and Shannon index. Beta diversity was assessed using Bray-Curtis and Jaccard distances. Relative abundances of dominant bacterial taxa were also determined. Sequencing was performed at the Department of Technology of FCAV/UNESP, Jaboticabal campus.

### Ciliated protozoa

Samples for the differential count of ciliated protozoa in the rumen were collected on day 20 of each experimental period, 4 hours after feeding. In this process, 10 mL of solid and liquid ruminal content collected for bacterial sequencing analysis were stored in tubes containing 20 mL of 50% formaldehyde (v/v). A total of 1 mL of the sample was diluted in formalin, with brilliant green and glycerol added, resulting in a 30-fold dilution. Protozoa were identified based on morphological characteristics, including cell size and shape, ciliation pattern, and nuclear and skeletal features, allowing classification into the genera *Isotricha*, *Dasytricha*, *Entodinium*, and *Diplodiniinae* subfamily, according to the taxonomic descriptions and identification keys proposed by Dehority [34]. The counted was using a Neubauer Improved Bright-Line counting chamber (Hausser Scientific Partnership R, Horsham, PA, United States) with internal dimensions of 50 mm x 20 mm x 1 mm (capacity 1 mL), as outlined by Dehority [35], where 100 optical fields were counted using a reticle.

### Ruminal papillae histology

Ruminal papillae samples were collected on day 21 of each experimental period. The rumen was temporarily emptied, and its contents were returned immediately after sampling. A portion of the ventral sac was exteriorized through the rumen fistula, and twelve papillae were randomly excised from a 2.5 × 2.5 cm area.

Samples were fixed, dehydrated, and embedded in paraffin wax. Tissue processing consisted of sequential immersion for 1 h in 70% ethanol, 90% ethanol, 100% ethanol, absolute ethanol I and II, xylene I and II, and paraffin I and II (Histosec®, Merck), followed by embedding in paraffin III (Histosec®), according to Odongo et al. [36]. Sections (6 µm) were obtained using a microtome and stained with hematoxylin and eosin.

Histological measurements included papillae height, width, and area, thickness of the keratinized epithelium, and mitotic index. Measurements were performed using a Leica Qwin Image Analyzer coupled to a Leica light microscope.

### Statistical analysis

Residual normality and heteroscedasticity were tested prior to analysis using Shapiro–Wilk and Kolmogorov–Smirnov tests; no data transformations were necessary. Treatment effects were considered fixed, while period, animal, and interactions were treated as random effects. Repeated measures over time were analyzed using the PROC MIXED procedure in SAS (SAS Inst. Inc., Cary, NC, USA) with the appropriate covariance structure selected based on Akaike and Schwarz’s Bayesian criteria. Linear and quadratic effects of narasin doses were evaluated as appropriate. Significance was declared at P < 0.05, and trends were discussed at P < 0.10. Bacterial sequencing data were processed using Mothur v.1.40.0 (www.mothur.org/wiki). Operational taxonomic units (OTUs) were clustered at 97% sequence similarity, and counts were normalized to 10,000 sequences per sample. Alpha diversity (within-sample diversity) was assessed using Chao1 species richness and Shannon diversity indices, while beta diversity (between-sample community composition) was analyzed using Bray–Curtis dissimilarity and visualized with non-metric multidimensional scaling (NMDS) in R (R Core Team). Differences in community structure were assessed using permutational multivariate ANOVA (PERMANOVA) with FDR-corrected pairwise comparisons.

## Results

### Intake and ruminal degradability in situ

A quadratic effect was observed on dry matter intake (DMI), expressed both in kg (P = 0.04) and as a percentage of body weight (P = 0.01). Including narasin in the diet of rumen-cannulated Angus cattle did not affect the degradability of DM, ash, CP, starch, NDF, or ADF. Different levels of narasin (13, 20, and 27 ppm) showed no significant impact on the ruminal degradation of these nutrients, with all effects being statistically non-significant (Table 2).

**Table 2:** Dry matter intake and in situ degradability of a feedlot diet with the addition of narasin provided to rumen-cannulated Angus cattle.

	Narasin (ppm)				*P* Value	
Item	13	20	27	SEM^1^	L^4^	Q^5^
Dry matter intake						
DMI, kg	20.70	20.19	23.47	0.86	0.01	**0.04**
DMI, kg/%BW	2.46	2.37	2.79	0.12	0.01	**0.01**
*In situ* degradability						
Dry Matter, %	60.54	60.00	61.52	3.01	0.37	0.19
Ash, %	74.68	74.97	74.79	1.28	0.89	0.79
NDF^2^, %	38.19	31.43	35.42	3.37	0.52	0.13
ADF^3^, %	21.30	21.96	22.03	4.09	0.70	0.85
Crude Protein, %	60.20	60.34	58.59	3.02	0.56	0.67
Starch, %	74.13	75.77	75.60	1.25	0.41	0.49

^1^Standard error of the mean; ^2^Neutral detergent fiber; ^3^Acid detergent fiber; ^4^ Linear effect; ^5^ Quadratic effect.

### Total dry matter and nutrient digestibility

Adding narasin to the diet of rumen-cannulated Angus cattle did not affect the total apparent digestibility of DM, ash, CP, or starch. However, a significant quadratic effect was found for the digestibility of ADF (P = 0.01), with 34.55%, 30.45%, and 38.05% for the 13, 20, and 27 ppm treatments, respectively (Table 3).

**Table 3:** Total apparent digestibility of a feedlot diet with the addition of narasin provided to rumen-cannulated Angus cattle.

	Narasin (ppm)				*P* Value	
Item	13	20	27	SEM^1^	Linear	Quadratic
Total Apparent Digestibility						
Dry Matter, %	83.60	83.55	83.45	0.08	0.20	0.82
Ash, %	61.94	70.22	58.27	6.51	0.69	0.22
NDF^2^, %	66.38	70.18	72.55	2.51	0.08	0.80
ADF^3^, %	34.55	30.45	38.05	2.01	0.14	**0.01**
Crude Protein, %	86.03	87.09	85.39	1.07	0.68	0.31
Starch, %	95.56	95.57	95.58	0.16	0.89	0.96

¹Standard error of mean; ^2^Neutral detergent fiber; ^3^Acid detergent fiber

### Continuous ruminal pH measurement

The results shown in Table 4 revealed a quadratic effect on DMI, measured in kg (P = 0.04) and as a percentage of BW (P = 0.01), and for mean pH (P = 0.04) with the lowest values found in cattle receiving 20 ppm of narasin. Moreover, a linear increase in minimum ruminal pH was identified as narasin supplementation increased (P = 0.05). In contrast, the time spent with ruminal pH below 5.2 and the area under pH 5.6 decreased linearly with higher narasin doses, indicating a beneficial effect on ruminal pH stability. Additionally, the duration of pH levels below 6.2 increased linearly as the doses of narasin rose (P = 0.07), and cattle consuming 20 ppm of narasin exhibited the largest pH area under 6.2 (P = 0.02).

**Table 4:** Dry matter intake, ruminal pH, temperature, and oxidation-reduction potential of rumen-cannulated Angus cattle fed feedlot diets supplemented with narasin.

	Narasin (ppm)				*P* Value	
Item	13	20	27	SEM^1^	Linear	Quadratic
Maximum pH	6.54	6.33	6.38	0.11	0.23	0.24
Minimum pH	5.39	5.44	5.70	0.14	**0.01**	0.19
Mean pH	5.93	5.75	5.97	0.12	0.73	**0.05**
pH < 5.2, min	160.83	73.33	0.00	84.51	**0.08**	0.76
pH < 5.6, min	333.30	429.00	10.00	170.29	0.12	0.13
pH < 6.2, min	980.00	1344.17	1240.83	82.33	**0.08**	0.76
pH area <6.2	462.19	662.12	358.04	161.26	0.85	**0.03**
pH area <5.6	146.03	101.05	0.65	82.33	**0.07**	0.36
pH area <5.2	52.05	8.30	0.00	16.90	0.12	0.81
Temperature, ºC	39.99	39.91	39.44	0.29	0.24	0.54
ORP^2^	−253.79	−253.79	−253.72	30.47	0.17	0.41
pH at 4h^3^	6.02	5.93	5.99	0.10	0.75	0.39

¹Standard error of mean; ²Ox-Redox Potential; ^3^post-feeding

### Evaluation of ruminal fermentation products

The results of ruminal fermentation products are presented in Table 5. Supplementation with 27 ppm of narasin led to lower concentrations of NH₃, acetate, and butyrate, as well as a reduction in total SCFA (P = 0.01). Cattle receiving 20 ppm of narasin showed a decrease in propionate concentration (P < 0.01) and a corresponding increase in the acetate-to-propionate ratio (P < 0.01). Across all three treatments, ruminal lactate concentration decreased quadratically (P = 0.03).

**Table 5:** Short-chain fatty acids profile, NH_3_, and lactate of rumen-cannulated Angus cattle fed feedlot diets supplemented with narasin.

	Narasin (ppm)				*P* Value	
Item	13	20	27	SEM^1^	L^3^	Q^4^
NH_3_, mg/dL	11.40	14.12	3.52	1.69	<0.01	**0.01**
Acetate, mmol/L	65.54	67.95	46.85	5.12	0.02	**0.01**
Propionate, mmol/L	49.93	27.04	35.78	4.52	0.12	**<0.01**
Butyrate, mmol/L	13.09	17.93	10.83	2.18	0.30	**<0.01**
Lactate, mmol/L	0.55	0.60	0.46	0.13	0.97	**0.03**
Total SCFA, mmol/L	128.72	111.36	93.15	8.86	**0.01**	0.94
ACET:PROP^2^	1.43	2.76	1.46	0.16	0.93	**<0.01**

¹Standard error of mean; ²Acetate to propionate ratio; ³Linear effect; ⁴Quadratic effect.

### Ruminal bacterial community sequencing

A total of 5,261,345 raw sequences were generated, with an average of 30,467 ± 2,301 sequences per sample (standard deviation). After quality control and filtering, samples contained an average of 4,467 unique OTUs, with Good’s coverage ≥ 0.89, indicating sufficient sequencing depth.

**Fig 1:**
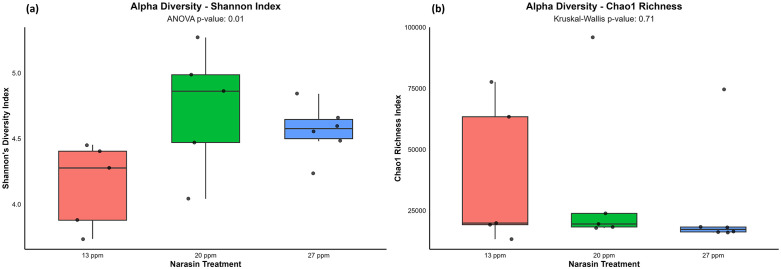
Alpha diversity of the ruminal microbiota in rumen-cannulated Angus cattle fed feedlot diets supplemented with narasin (13, 20, and 27 ppm). (a) Shannon diversity index and (b) Chao richness index. Boxplots represent the median, interquartile range, and minimum and maximum values, while dots indicate individual observations. *P* values denote the overall treatment effect.

Alpha bacterial diversity assessed by Shannon’s index exhibited a significant quadratic response to narasin supplementation (*P* = 0.01), with the highest diversity observed in animals receiving 20 ppm of narasin, whereas both lower (13 ppm) and higher (27 ppm) inclusion levels resulted in reduced diversity (Fig 1A). In contrast, bacterial richness estimated by the Chao index was not affected by narasin supplementation (*P* = 0.71; Fig 1B).

**Fig 2:**
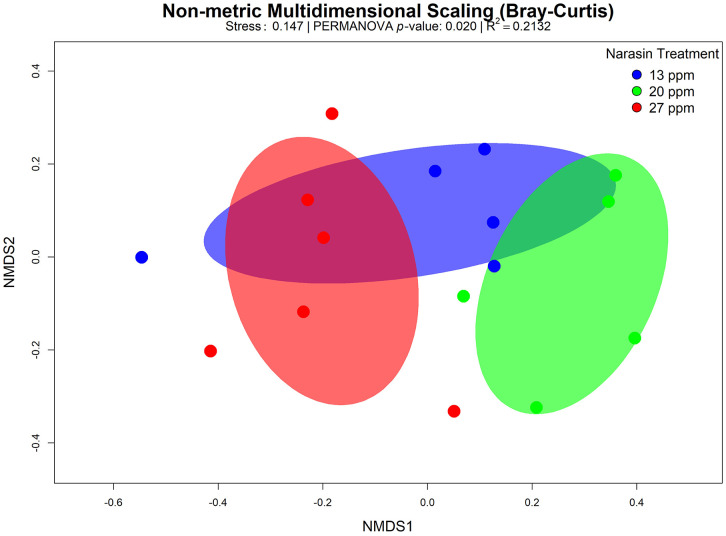
Non-metric multidimensional scaling (NMDS) ordination based on Bray–Curtis dissimilarity of ruminal bacterial community composition in rumen-cannulated Angus cattle fed feedlot diets supplemented with narasin (13, 20, and 27 ppm). Each point represents an individual sample, and ellipses indicate the 95% confidence intervals for each dietary treatment. Community dissimilarity was evaluated by PERMANOVA (*P* = 0.02; R² = 0.2132). The stress value of the NMDS ordination was 0.147.

The non-metric multidimensional scaling (NMDS) ordination revealed a partial separation of the ruminal bacterial community structure among narasin supplementation levels. Samples from animals receiving 20 ppm of narasin tended to cluster apart from those fed 13 ppm, indicating a shift in overall community composition associated with the intermediate supplementation level. In contrast, samples from the 27-ppm treatment showed greater dispersion and overlapped with both the 13 and 20 ppm groups, suggesting increased within-treatment variability and a less consistent compositional response at the highest narasin inclusion level. Overall, these patterns indicate that narasin supplementation modulated ruminal bacterial beta diversity, with the most distinct community structure observed at 20 ppm, whereas lower and higher doses resulted in more overlapping microbial profiles. (Fig 2)

**Fig 3:**
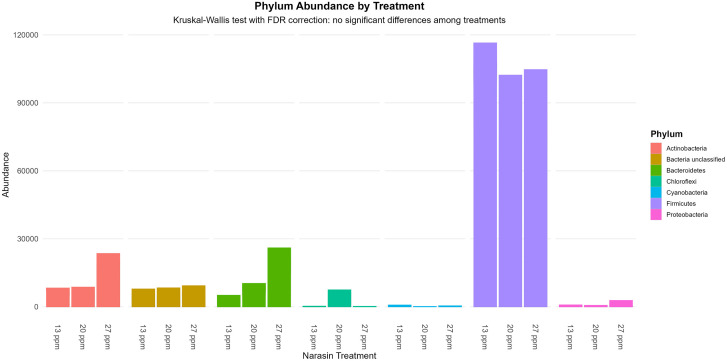
The seven most abundant phyla of ruminal bacteria in rumen-cannulated Angus cattle fed feedlot diets supplemented with narasin (13, 20, and 27 ppm). Statistical differences among treatments were tested using the Kruskal–Wallis test followed by false discovery rate (FDR) correction. No significant differences were detected (FDR-adjusted *P* > 0.05).

**Fig 4:**
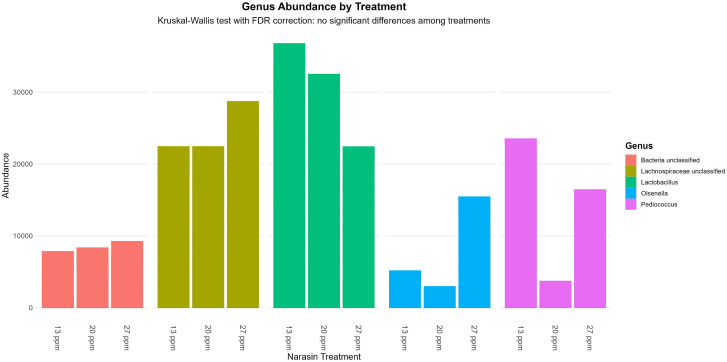
The five most abundant ruminal bacterial genera in rumen-cannulated Angus cattle fed feedlot diets supplemented with narasin (13, 20, and 27 ppm). Statistical differences among treatments were tested using the Kruskal–Wallis test followed by false discovery rate (FDR) correction. No significant differences were detected (FDR-adjusted *P* > 0.05).

The relative abundance of the five most prevalent bacterial genera and the seven most abundant phyla is presented in Figs 3 and 4, respectively. Firmicutes was the dominant phylum across treatments (Fig 3), and members of the genus *Lactobacillus* represented the most abundant lactic acid–producing bacteria. Although some visual differences in the relative abundance of specific taxa were observed among narasin treatments, particularly a tendency for lower abundance of lactic acid–producing bacteria at 20 and 27 ppm (Fig 4), no significant differences were detected after Kruskal–Wallis testing with FDR correction (*P* > 0.05).

**Fig 5:**
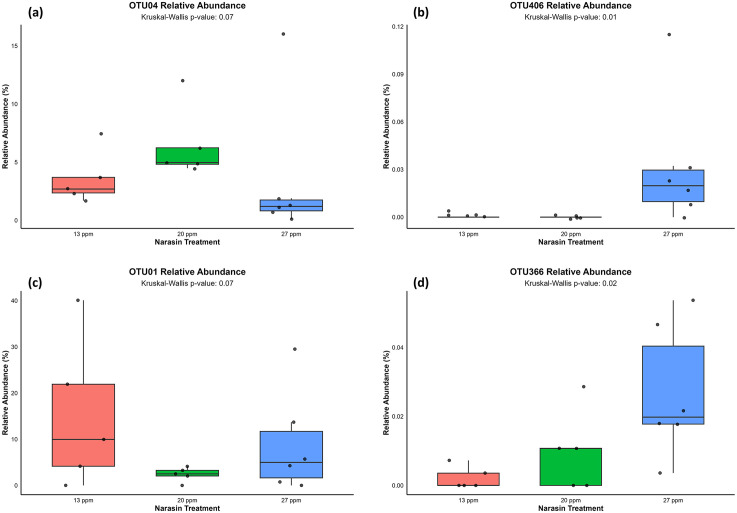
Relative abundance of selected ruminal bacterial taxa at the OTU level in rumen-cannulated Angus cattle fed feedlot diets supplemented with narasin (13, 20, and 27 ppm). (A) Lachnospiraceae family (OTU04); (B) *Megasphaera* genus (OTU406); (C) *Pediococcus acidilactici* (OTU001); and (D) ***Selenomonas ruminantium* (OTU366).**

At the OTU level, narasin supplementation elicited distinct quadratic responses in specific bacterial taxa (Fig 5). Members of the Lachnospiraceae family, tended exhibited a quadratic response, with the greatest relative abundance observed in animals receiving 20 ppm of narasin (*P* = 0.07; Fig 5A). *Megasphaera spp*. showed a linear pattern across treatments, with higher abundance at 27-ppm of narasin level (*P* = 0.01; Fig 5B). A tendency a lower abundance of *Pediococcus acidilactici* was observed in animals supplemented with 20 ppm of narasin (quadratic *P* = 0.07; Fig 5C). The abundance of *Selenomonas ruminantium* was significantly influenced by narasin supplementation, exhibiting a clear linear increase with increasing narasin inclusion levels (*P* = 0.02; Fig 5D).

### Ciliated protozoa

In Table 6, a linear increase was observed (P = 0.04) in the number of Isotricha as the narasin dose increased. It is worth noting that the Isotricha genus is more prevalent under higher ruminal pH conditions. Additionally, cattle fed 20 ppm of narasin had the highest number of protozoa in the rumen environment.

**Table 6:** Differential protozoa count of rumen-cannulated Angus cattle fed feedlot diets supplemented with narasin.

	Narasin (ppm)				*P* Value	
Item	13	20	27	SEM^1^	Linear	Quadratic
*Dasytricha* x 10^3^/ml	0.17	0.1	0.68	0.23	0.14	0.16
*Isotricha* x 10^3^/ml	1.05	1.96	3.39	0.87	**0.04**	0.77
*Entodinium* x 10^3^/ml	62.27	61.98	55.61	7.34	0.17	0.45
*Diplodinium* x 10^3^/ml	36.51	36.06	40.33	7.42	0.41	0.55
Total x 10^3^/ml	165.4	194.8	142.8	15.38	0.24	**0.02**

¹Standard error of the mean

### Ruminal papillae histology

The results from Table 7 on ruminal morphometry for rumen-cannulated Angus cattle fed feedlot diets supplemented with narasin indicate significant effects on certain parameters. Papillae width increased significantly with higher narasin levels (*P* < 0.01). Similarly, the papillae area showed a significant linear increase (*P* < 0.01). A significant quadratic effect was observed for keratin layer thickness (*P* = 0.02). No significant effects were found for papilla height, mitotic index (n), or mitotic index (%).

**Table 7:** Ruminal histology for rumen-cannulated Angus cattle fed feedlot diets supplemented with narasin.

	Narasin (ppm)				*P* Value	
Item	13	20	27	SEM^1^	Linear	Quadratic
Papillae height, mm	10.20	10.51	11.15	0.58	0.24	0.81
Papillae width, mm	0.28	0.35	0.40	0.02	**<0.01**	0.44
Papillae area, mm^2^	2.80	3.68	4.56	0.35	**<0.01**	0.99
KLT^3^, um	8.12	6.40	7.27	0.40	0.16	**0.02**
Mitotic index, n	49.00	45.50	45.50	2.14	0.26	0.51
Mitotic index, %	2.45	2.28	2.28	0.11	0.26	0.51

^1^Standard error of the mean; ^2^Keratin layer thickness

## Discussion

Narasin supplementation has been extensively researched because of its effects on the rumen microbiota, leading to significant modifications in bacterial composition and fermentative dynamics within the rumen. Studies indicate that narasin favors the selection of Gram-negative bacteria and directly influences microbial diversity and ruminal fermentation profiles [2,5,15]. Among the evaluated doses in this study, 20 ppm promoted greater ruminal microbial diversity (Shannon index) and enhanced pH stability. While these changes indicate a more balanced microbiome, they did not necessarily translate into increased fermentative efficiency, as SCFA and propionate concentrations did not differ in a way that supports this conclusion. These findings are consistent with previous studies indicating that ionophores modulate the microbiota by promoting bacteria that utilize lactate and propionate, which reduces excessive lactic acid production and, subsequently, the risk of ruminal acidosis [37,38].

The influence of narasin on bacterial composition is notable, particularly regarding the increased abundance of Lachnospiraceae, a family of butyrate-producing bacteria that generate volatile fatty acids essential for maintaining the integrity of the ruminal epithelium and stabilizing pH levels [39,40]. Butyrate is recognized for stimulating the growth and differentiation of ruminal epithelial cells, which enhances SCFA absorption and supports nutrient utilization [41,42]. From a functional perspective, this shift suggests that narasin contributes to a ruminal environment that supports epithelial health. Furthermore, higher doses of narasin significantly reduced the population of lactic acid-producing bacteria, such as *Lactobacillus* and *Pediococcus*, which are known to contribute to ruminal acidosis when present in excess [43,44]. This reduction has practical implications for feedlot cattle, as it may lower the risk of subacute ruminal acidosis and promote greater ruminal stability. Taken together, these microbial shifts indicate that narasin supplementation not only alters bacterial composition but also contributes to maintaining ruminal health.

The 13-ppm dose promoted increased total SCFA production and propionate. Propionate is a vital precursor of hepatic gluconeogenesis and is directly associated with feed efficiency in ruminants [45]. On the other hand, a 20-ppm dose increased the acetate and lactate production and increased the acetate:propionate ratio. These findings complement previous research and enhance our understanding of the effects of narasin on feedlot performance and carcass characteristics in Nellore cattle, as noted in the companion study by Silva et al. [14], where cattle consuming 20 ppm of narasin exhibited higher fat deposition at the 12th rib, which may be associated with enhanced ruminal stability, as observed in this study, and decreased DMI when cattle consumed 20 ppm of narasin. In addition, this greater stability supports the findings of the present study, in which animals fed 20 ppm of narasin exhibited increased concentrations of acetate, butyrate, and NH₃, alongside a decrease in propionate concentration and a reduced duration of pH < 5.6. Elevated acetate levels serve as a primary precursor for lipogenesis in ruminants, being efficiently converted into acetyl-CoA and subsequently into fatty acids in adipose tissue [46]. The increased availability of acetate likely contributed to enhanced lipogenesis, promoting fat deposition in the carcass, as reported in the companion study by Silva et al. [14]. Moreover, the improved ruminal pH stability may have prevented subclinical acidosis, allowing for more consistent microbial fermentation and efficient nutrient utilization.

The dose of 27 ppm of narasin led to reduced ammonia and total SCFA production. This effect may be related to the selection of less efficient microorganisms in SCFA production and the inhibition of proteolytic microorganisms responsible for protein degradation and NH_3_ release [47]. The diversity of bacterial communities increased when the narasin dose rose from 13 to 20 ppm. Additionally, based on the Bray-Curtis dissimilarity test, the bacterial communities of 27-ppm cattle were significantly different from the microbiota of Nellore cattle fed 13 ppm of narasin.

Rumen pH stability is crucial for cattle health and performance. Doses of 20 and 27 ppm of narasin provided enhanced pH stability over time, reducing episodes of excessive acidification and promoting the survival of ruminal protozoa, such as *Isotricha*, which play a vital role in fiber degradation [35,48]. Ruminal pH stability was evaluated by measuring the cumulative time the pH remained below the critical threshold of 5.2. Supplementation with narasin reduced the duration below this pH, indicating a lower risk of ruminal acidosis. Maintaining the pH within the optimal range supports fiber digestion, preserves microbial balance, and enhances overall ruminal fermentation [49].

The relationship between ruminal pH and protozoa is also evident. The 20-ppm dose resulted in the highest total number of protozoa, while the 27-ppm dose specifically increased *Isotricha*, contributing to enhanced acid-detergent fiber (ADF) digestibility. The linear increase of *Isotricha* and the reduction of total protozoa at 27 ppm may have contributed to the lower NH₃ concentration observed in this treatment, as ruminal protozoa are known to engage in bacterial predation and protein degradation, thereby contributing to intraruminal nitrogen cycling and ammonia release. This functional role of protozoa in nitrogen metabolism has been reported in recent studies of rumen microbial ecology and nitrogen utilization in ruminants [50]. Concurrently, the maintenance of an active *Isotricha* population, which prefers environments with higher pH, likely aided in fiber digestion and stabilization of ruminal pH, favoring the higher ADF digestibility observed in animals supplemented with 27 ppm. Thus, the data suggest that the total reduction of protozoa may partially explain the decrease in NH_3_, while the persistence of specific protozoa contributes to efficient fiber fermentation and maintenance of a stable ruminal environment, integrating effects on pH, NH_3_, and digestibility [51,52].

The increased DMI was also associated with better ADF digestibility, as efficient fermentation accelerates the ruminal passage rate, promoting greater DMI. Studies indicate that a more balanced microbiome is related to improved fiber utilization, resulting in enhanced feed efficiency [53,54]. Although the modulation of the microbiota and fermentation profile showed significant impacts from the use of different narasin doses, ruminal degradability was not influenced by the dosages evaluated in this study.

It’s important to note that the impact of narasin on the ruminal epithelium also warrants attention. The increased dosage was linked to wider papillae and, as a result, a larger absorption area. Interestingly, the 27-ppm dose exhibited the greatest width and area of papillae, despite showing the lowest butyrate concentration. This suggests that the development of papillae may be more related to a more stable ruminal pH and a less aggressive ruminal environment, which could favor epithelial morphology. These findings emphasize the significance of ruminal stability for the proper development of ruminal epithelium [39,40].

Due to the higher amount of antimicrobial substances, the 20-ppm dose appears to promote greater ruminal stability than the 13-ppm dose, although it results in lower ruminal VFA and propionate concentrations. In intensive beef systems with high-quality feeding management, the 13-ppm dose may be more suitable. However, if the nutritionist seeks greater assurance to counteract poor feeding management, the 20-ppm dose appears to be the better option, including a reduction in DMI when compared to the 13-ppm dose.

In summary, supplementing with narasin significantly affects the fermentation dynamics and structure of the ruminal microbial ecosystem, influencing the production of SCFA and maintaining pH stability.

## Conclusion

Different doses of narasin supplementation effectively enhance ruminal pH stability, microbial diversity, and fermentation efficiency in Angus cattle on feedlot diets. The findings of this study recommend a narasin dose between 13 and 20 ppm for such cattle under these conditions. The 20-ppm dose was associated with greater ruminal stability, whereas the 13-ppm dose proved to be effective in increasing concentrations of propionate and SCFA. For nutritionists looking to offer additional safeguards against ruminal acidosis, 20 ppm remains the preferred choice. Therefore, both doses present viable strategies depending on the nutritional context, and the specific management and dietary conditions of the feedlot system should guide their use.
